# Performance of a Real-Time PCR Assay for the Detection of Five *Candida* Species in Blood Samples from ICU Patients at Risk of Candidemia

**DOI:** 10.3390/jof9060635

**Published:** 2023-05-31

**Authors:** Gabriel N. Felix, Vera L. T. de Freitas, Afonso R. da Silva Junior, Marcello M. C. Magri, Flavia Rossi, Odeli N. E. Sejas, Edson Abdala, Luiz M. S. Malbouisson, Thais Guimarães, Gil Benard, Gilda M. B. Del Negro

**Affiliations:** 1 Laboratory of Medical Mycology (LIM 53), Instituto de Medicina Tropical, Hospital das Clínicas da Faculdade de Medicina da Universidade de São Paulo (HC-FMUSP), São Paulo 05403-000, Brazil; gnavesf@gmail.com (G.N.F.); bengil60@gmail.com (G.B.); 2 Laboratório de Investigação Médica em Imunologia (LIM 48), Hospital das Clínicas da Faculdade de Medicina da Universidade de São Paulo (HC-FMUSP), São Paulo 05403-000, Brazil; vera_lucia@yahoo.com; 3 Central Laboratory Division (LIM 03), Hospital das Clínicas da Faculdade de Medicina da Universidade de São Paulo (HC-FMUSP), São Paulo 05403-010, Brazil; rafael.biomedicina11@gmail.com (A.R.d.S.J.); marcello.magri@hc.fm.usp.br (M.M.C.M.); f.rossi@hc.fm.usp.br (F.R.); 4 Departamento de Moléstias Infecciosas e Parasitárias, Faculdade de Medicina, Universidade de São Paulo, São Paulo 05403-010, Brazil; 5 Cancer Institute of São Paulo State (ICESP), Hospital das Clínicas da Faculdade de Medicina da Universidade de São Paulo (HC-FMUSP), São Paulo 01246-000, Brazil; odeli.sejas@hc.fm.usp.br (O.N.E.S.); edson.abdala@hc.fm.usp.br (E.A.); 6 Discipline of Anesthesiology, Faculdade de Medicina, Universidade de São Paulo, São Paulo 05403-010, Brazil; luiz.malbouisson@hc.fm.usp.br; 7 Infectious Diseases Department, Hospital do Servidor Público Estadual de São Paulo (IAMSPE), São Paulo 04029-000, Brazil; thais.guimaraes@hc.fm.usp.br

**Keywords:** molecular diagnosis, real-time PCR, candidemia, *Candida*, 1,3-β-D-glucan

## Abstract

The gold standard for diagnosing invasive candidiasis still relies on blood cultures, which are inefficient and time-consuming to analyze. We developed an in-house qPCR assay to identify the 5 major *Candida* species in 78 peripheral blood (PB) samples from ICU patients at risk of candidemia. Blood cultures and (1,3)-β-D-glucan (BDG) testing were performed concurrently to evaluate the performance of the qPCR. The qPCR was positive for DNA samples from all 20 patients with proven candidemia (positive PB cultures), showing complete concordance with *Candida* species identification in blood cultures, except for detection of dual candidemia in 4 patients, which was missed by blood cultures. Additionally, the qPCR detected *Candida* species in six DNA samples from patients with positive central venous catheters blood (CB) but negative PB cultures. BDG values were similarly high in these six samples and the ones with proven candidemia, strongly suggesting the diagnosis of a true candidemia episode despite the negative PB cultures. Samples from patients neither infected nor colonized yielded negative results in both the qPCR and BDG testing. Our qPCR assay was at least as sensitive as blood cultures, but with a shorter turnaround time. Furthermore, negative results from the qPCR provided strong evidence for the absence of candidemia caused by the five major *Candida* species.

## 1. Introduction

The occurrence of invasive candidiasis (IC) continues to pose a serious threat in nosocomial settings, with candidemia episodes being the most frequent manifestation. Candidemia is particularly common among critically ill patients in the ICU, with significant morbidity and mortality rates. Approximately 45% of all nosocomial candidemia episodes occur in this population [1,2,3]. Sepsis and septic shock may occur in about 30% to 40% of patients with *Candida* hematogenous infections [4]. The most common risk factors for invasive candidiasis typically include prolonged administration of broad-spectrum antibiotics, major abdominal surgery, long-term ICU stay, steroid treatment, central venous catheters, parenteral nutrition, and hemodialysis [5,6].

The risk of mortality and hospitalization costs increase with delayed initiation of appropriate antifungal therapy, making early and accurate diagnosis of infections caused by *Candida* species highly desirable. However, this still presents a challenge for patients with candidemia [1,7,8]. Furthermore, the overuse of antifungal therapy due to difficulties in establishing a correct diagnosis can lead to the emergence of resistant *Candida* strains in nosocomial environments [5].

*Candida albicans*, *Candida glabrata*, *Candida parapsilosis* complex, *Candida tropicalis*, and *Candida krusei* represent approximately 95% of the species isolated from candidemia episodes worldwide [5,9]. In Brazil, a recent retrospective study conducted in public tertiary hospitals showed that these five *Candida* species were responsible for 95.7% of the candidemia episodes. The remaining 5% corresponded to a variety of other *Candida* species that are infrequently identified as causative agents of invasive disease [10].

The isolation of yeasts from blood cultures remains the gold standard for diagnosing IC. However, its overall sensitivity rate in the spectrum of IC is on average only 50%, and further decreases in the presence of antifungal drugs. Moreover, the time period (48–72 h or longer) required for the identification of *Candida* species limits early diagnosis, which can impact the management of critically ill patients in the ICU [4,11,12]. On the other hand, recovering the infecting yeasts by culture allows for drug susceptibility testing [11].

The mannan antigen (MnAg) and the anti-mannan antibody (MnAb) have been used for diagnosing hematogenous *Candida* infections. However, both sensitivity and specificity values of MnAg and MnAb are quite variable, ranging from 31% to 100% and from 44% to 100%, respectively. Additionally, these values also vary among *Candida* species, with higher values observed for *C. albicans*. Although it has been proposed that combined MnAg/MnAb detection would increase sensitivity and specificity [13,14], a recent study found poor prognostic values of MnAg and MnAb, alone or in combination, for the occurrence of invasive candidiasis in high-risk non-immunocompromised ICU patients [15].

To overcome the limitations of the aforementioned *Candida* diagnostic methods, PCR-based assays for detecting yeast DNA in clinical specimens have been extensively explored in recent decades [16,17,18,19,20,21]. Several in-house assays have been evaluated, employing panfungal or *Candida* species-specific primers, different DNA extraction methods from various sample types, and diverse types of platforms. As a result, the performance of these in-house molecular assays has provided variable results, with sensitivity and specificity values ranging from 56.2% to 100% and from 54% to 100%, respectively [22,23,24]. In the clinical context, these assays also present heterogenous positive predictive values, depending on the prevalence of candidemia in the specific nosocomial setting. Nevertheless, the negative predictive values tend to be high, making these assays more useful for ruling out hematogenous infections caused by *Candida* species, thereby allowing for early discontinuation of prophylactic or empirical antifungal therapy [7,25]. However, the lack of standardization and validation of these techniques hampers their application in routine laboratory diagnosis [26].

A number of commercial tests are available, but no evidence of superiority over in-house PCR assays has been demonstrated. Most of them have not yet been validated in multicenter studies [27]. The T2 Candida panel is the only commercial kit approved by the Food and Drug Administration (FDA) so far, providing rapid results with a low limit of detection [21,23]. The test has shown good performance, particularly when spiked blood samples are used, with sensitivity and specificity of 91.15% and 99.4%, respectively [28]. However, follow-up studies of patients with proven *Candida* infection (some of whom were also receiving antifungal drugs) showed sensitivity values of less than 50% [21,29]. In addition, the high cost of the assay makes it unaffordable for health services in developing countries, as each individual sample test costs around US$300, and the equipment is also expensive [7,24,29].

The BDG serological assay has been widely used as an auxiliary test for diagnosing candidemia [30,31] due to its low specificity, as increased BDG serum levels can also be detected in other fungal infections [32]. Previous studies have evaluated the performance of the BDG assay for diagnosing invasive candidiasis, resulting in variable sensitivity (ranging from 65% to 93%) and specificity (74% to 94%) values, as well as positive and negative predictive values (42% to 68% and 77% to 99%, respectively) [32,33,34,35,36]. Subsequently, high negative predictive values have consistently been demonstrated in many reports, with the BDG assay mainly assisting clinicians in deciding whether or not to discontinue antifungal therapy in presumed cases [33,34,37]. Furthermore, the use of BDG in conjunction with PCR assays has improved the sensitivity for diagnosing candidemia [38,39]. Unfortunately, due to its high cost, the BDG test is not available for routine screening in public or private laboratories in Brazil, nor in other developing countries.

In summary, the current methods for early diagnosis of IC are limited by low sensitivity and delayed results. Molecular-based techniques, such as real-time PCR (qPCR), hold promise, but further studies are needed for their validation and incorporation into routine laboratory diagnosis and clinical practice [7,8]. This is the reason why, in this study, the performance of a qPCR assay was evaluated for the detection and identification of the main clinically relevant *Candida* species in PB samples from suspected ICU candidemia patients, who are the most important group at risk for episodes of candidemia, which in turn are associated with high mortality rates [4]. Parallel testing with blood cultures evaluated by the BDG test were also conducted, to assess the performance of the qPCR assay.

## 2. Materials and Methods

### 2.1. Patients and Clinical Samples

This prospective multicentric study was conducted between May 2018 and March 2020 in the Adult Intensive Care Units (AICU) of three tertiary care, teaching university hospitals in São Paulo city, Brazil. Patients who presented clinical signs of *Candida* infection—such as fever unresponsive to broad-spectrum antibacterial treatment; signs suggestive of sepsis; AICU stay of more than 48 h; and presence of risk factors including abdominal surgery, central vein catheter (CVC), total parenteral nutrition, dialysis, steroid treatment, and immunosuppressive treatment—were eligible for inclusion in the study [4]. Exclusion criteria included age of less than 18 years, severe neutropenia, human immunodeficiency virus infection, prior diagnosis of other systemic fungal infections (especially aspergillosis, mucormycosis, and pneumocystosis), antifungal treatment before admission to AICU, and refusal to sign the informed consent. Data on demographic and clinical characteristics, presence of risk factors, and outcomes were obtained from medical records for each patient. The study was approved by the ethics committees of all institutions involved.

In this study, PB samples were collected simultaneously for fungal culture, qPCR, and BDG test at the moment of clinical suspicion of *Candida* infection and prior to antifungal prescription. CB cultures were routinely collected by the AICU staff before antifungal prescription. Written informed consent was obtained from all patients or their legal representatives.

### 2.2. Blood Cultures and MALDI–TOF MS for Identification of Candida Isolates

Cultures were obtained by inoculating 10 mL of PB and CB samples into Bactec Plus Aerobic/F™ and Plus Anaerobic/F™ bottles (BD, Franklin Lakes, NJ, USA), which were then incubated in the Bactec System^TM^ (BD) for up to seven days. 

MALDI–TOF MS technique was carried out directly from the blood culture bottles on the same day that the Bactec System yielded a positive result. Two milliliter aliquots collected from each bottle were subjected to a previously described in-house protein extraction protocol [40]. The spectra were acquired using the Vitek^®^ MS platform (BioMérieux, Marcy-l’Etoile, France) and analyzed using the SARAMIS™ database. In parallel, the content of the positive bottles was seeded in BD^TM^ CHROMagar^TM^ Candida Medium (Becton Dickinson, Sparks, MD, USA) and colonies were identified using MALDI–TOF MS, according to the laboratory’s standard protocols.

### 2.3. BDG Detection in Serum Samples

The BDG detection was performed in serum samples using the Fungitell^®^ assay, which is a commercially available kit (Associates of Cape Cod., East Falmouth, MA, USA). The assay was conducted according to the manufacturer’s protocol. Serum samples were analyzed in duplicate, and the mean value of the duplicates was used for further analysis. The positivity cutoff for the assay was set at ≥80 pg/mL, as per the manufacturer’s instructions. If the BDG results were higher than 523 pg/mL, no further dilutions were carried out.

### 2.4. DNA Extractions and qPCR Assays

DNA samples were obtained from 3 mL of PB collected using EDTA Vacutainer^®^ tubes. The Rapid Pure DNA Tissue Kit (MP Biomedical, Illkirch Cedex, France) was used for DNA extraction. Initially, PB samples were mixed with a lysis solution to remove hemoglobin, and then cell pellets were subjected to sonication using the Fast Prep-24 5G instrument (MP Biomedical). After lysis, but before DNA extraction, the samples were spiked with 1 × 10^4^ plasmids containing a sequence coding for gibberellin 2-beta-dioxygenase from *A. thaliana*, which served as an internal control to monitor the efficiency of DNA extraction [41,42]. The remaining steps of DNA extraction were carried out following the manufacturer kit’s protocol. The concentration of the DNA samples was estimated using a UV spectrophotometer Nanodrop LITE (Thermo Fisher Scientific, Wilmington, DE, USA).

The qPCR was performed using an intercalant DNA dye. A volume of 20 μL of reaction mixture containing 10 μL of 2X Power SYBR™ Green PCR Master Mix (Applied Biosystems, Carlsbad, CA, USA), 0.25 μM of each primer (as listed in Table S1), and 5 µL containing 100 ng of DNA from clinical samples was used for the amplifications in the ABI Step One Plus Real-Time PCR System (Applied Biosystems). An individual mixture reaction was performed with each of the five species-specific primers, as well as for the internal control.

The PCR conditions were as follows: an initial denaturation step of 95 °C for 10 min, followed by 40 cycles of 95 °C for 30 s, 62 °C for 30 s, and 72 °C for 30 s. Melt curves were generated by heating from 62 to 95 °C, with increments of 0.3 °C/s. All experiments were run in duplicate, and standard curves and negative controls were included in each qPCR assay.

The ITS regions from rRNA gene of *C. albicans* (ATCC 90028), *C. glabrata* (ATCC 90050), *C. parapsilosis* complex [*C. parapsilosis* sensu stricto (ATCC 22019), *C. orthopsilosis* (ATCC 96141), *C. metapsilosis* (ATCC 96143)], *C. tropicalis* (ATCC 750), and *C. krusei* (ATCC 6258) were amplified using previously described primers (Table S1). The resulting PCR products were cloned with the CloneJET PCR Cloning Kit (Thermo Fisher Scientific, Wilmington, DE, USA). Standard curves were generated with serial 10-fold dilutions of the recombinant plasmids of the five *Candida* species, ranging from 10^5^ to 10^0^ copies/reaction, in 20 independent experiments. The limit of detection (LoD) was determined as the lowest number of copies detected in ≥95% of the experiments [43]. To assess potential interference from human DNA in clinical samples, blood samples from healthy human donors were serially spiked with each of the five *Candida* recombinant plasmids to obtain 10^5^ to 10^0^ copies/mL. The spiked samples were then subjected to DNA extraction and analyzed in the molecular assay in three independent experiments.

To confirm the specificity of the qPCR assay, DNA samples from other *Candida* species, including *Candida lusitaniae*, *Candida kefyr*, *Candida famata*, *Candida guilliermondii*, and *Candida dubliniensis*, as well as DNA samples from other opportunistic fungi such as *Trichosporon* sp., *Cryptococcus* sp., *Rhodotorula* sp., *Aspergillus* sp., and *Fusarium* sp., and bacteria including *Acinetobacter baumannii*, *Pseudomonas aeruginosa*, *Staphylococcus aureus*, *Streptococcus pneumoniae*, and *Klebsiella pneumoniae* were evaluated. Additionally, DNA samples from healthy human subjects were also included for analysis. Analytical specificity was further assessed by testing each *Candida* species-specific primer against yeasts DNA and blood samples spiked with plasmids from the other four heterologous species.

### 2.5. Statistical Analysis

The data analysis was conducted using SPSS for Windows, Version 24.0 (SPSS Inc., Chicago, IL, USA). Categorical parameters were expressed as percentages, while continuous variables were summarized as medians and interquartile ranges (IQRs).

To assess the concordance between the qPCR results and blood cultures, as well as between the qPCR results and BDG assay, the kappa index of concordance was calculated.

## 3. Results

### 3.1. Patients

A total of 95 patients admitted to the AICU with clinical suspicion of candidemia were initially enrolled during the study period. From this, 17 patients (17.9%) were excluded from the analysis due to various reasons, including lack of simultaneous sample collection for blood culture, qPCR, and BDG assays *(n* = 7); patients starting antifungal treatment more than 48 h prior to blood sampling (*n* = 5); legal representatives of patients denying signing the consent form (*n* = 4); and 1 patient with a diagnosis of aspergillosis (*n* = 1).

The demographic characteristics, clinical conditions, and outcomes of the remaining 78 study patients are summarized in Table 1. Briefly, out of these 78 patients, 40 (51.2%) were male, with a median age of 60 years. The most frequent underlying disease was neoplasia, accounting for 66.6% of cases, with 90% of them being solid tumors. All patients had a central venous catheter (CVC) in place and were receiving broad-spectrum antibiotic therapy for more than 96 h at the time of candidemia suspicion. Fifty-seven patients (73.1%) presented previous *Candida* spp. colonization in at least one of the following anatomical sites: CVC, urinary catheter, and tracheal fluid. The 30-day mortality rate was 48.7%, with a median length of AICU stay of 12.5 days (IQR 8.0 and 25.0).

### 3.2. Analytical Performance of the qPCR

The LoD of the qPCR assay using SYBR™ Green reagent was determined to be 10 copies of the recombinant plasmids per assay, based on the standard curve experiments. This corresponded to a mean Cq value of 34.3 (range 32.8–35.6) ± 1.1 (range 0.7–1.6) for all five *Candida* species. The LoD obtained with spiked blood samples was also found to be the same, indicating that human DNA did not interfere with the efficiency of the assay. Samples with Cq values ≤ 36.0 were considered positive based on these results.

Regarding the analytical specificity, DNA samples from bacteria, non-*Candida* yeasts, all *Candida* species outside the selected major ones, and from healthy individuals did not produce any amplification signal. Of note, no cross-reactivity was detected when each *Candida* species primers was tested against yeast DNA and blood samples spiked with plasmid DNA from the other four heterologous species.

The Cq values obtained by amplification of *A. thaliana* gibberellin sequence (1 × 10^4^ copies) added to patients’ samples before DNA extractions ranged from 18 to 20 in all assays with the clinical samples, indicating that the DNA extraction protocol was effective.

### 3.3. Blood Cultures, qPCR, and BDG Assays in Patients’ Samples

Out of the 78 patients included in the study, 20 patients had positive PB cultures for *Candida* species, confirming the presence of candidemia. The time to yield positive results from PB cultures ranged from 24 to 72 h. Among the *Candida* species isolated from PB cultures, *C. albicans* was the most common, isolated from 35.0% of the PB samples, followed by *C. parapsilosis* from 30.0%, *C. tropicalis* from 20.0%, and *C. glabrata* from 15.0%. No *C. krusei* was isolated in this study.

MALDI–TOF MS performed directly from the positive bottles was concordant with all results of PB cultures for *Candida* species identification, except in two instances where indeterminate results were observed due to the concomitant presence of bacteria (*S. epidermidis* and *E. faecium*) in the patients’ PB samples. Similarly, MALDI–TOF MS provided 100% concordant results with the *Candida* species identification from CB cultures.

The molecular assay was positive for all DNA samples from the 20 patients with candidemia, with Cq values ranging from 17.8 to 35.8 (Table 2). The qPCR assay not only showed 100% concordance with the species identification of PB cultures, but also identified a second species of *Candida* in four samples that was missed by PB cultures (Table 2). The BDG test was positive with high values in all samples, except for one sample from a patient with single *C. parapsilosis* infection (Table 2). In this group, the median serum BDG concentration was 478.1 pg/mL (IQR 235.3 and 523.0).

Out of the 58 patients with negative PB cultures, 44 had positive cultures from either a CB sample (n = 32), an indwelling urologic device with >100,000 CFU/mL (n = 10), or tracheal fluid with >500,000 CFU/mL (n = 2), collected up to six days prior to inclusion in the study. *Candida parapsilosis* was the most frequently isolated species from the CB samples (50.0%), followed by *C. albicans* (18.7%), while *C. albicans* was the predominant species (90%) isolated from the urinary tract. The qPCR assay detected *Candida* DNA in samples from six patients, all of whom had positive CB cultures (Table 2). The species identified by the molecular technique in the PB samples were the same as the *Candida* species isolated from the CB cultures, except in one case where *C. krusei* DNA was detected by qPCR while the CB culture identified *C. parapsilosis* (sample S24, Table 2). Interestingly, indicating a true candidemia episode, five out of these six samples had very high BDG levels (median of 457.0 pg/mL, IQR 290.7 and 523.0), with the negative result coming from the PB sample in which *C. parapsilosis* DNA was detected (S15, Table 2). Of note, the median BDG value of the other 26 samples from patients with positive CB cultures and negative qPCR was lower (131.2 pg/mL, IQR 31.7 and 355.0) than that of the six samples with positive qPCR, although the difference did not reach statistical significance (*p* = 0.054). Similarly, the median BDG value in samples from the urinary tract- or tracheal fluid-colonized patients was also significantly lower than that of the six samples with positive qPCR (62.5 pg/mL, IQR 29.6 and 104.3, *p* = 0.008).

Lastly, the qPCR assay was consistently negative in the DNA samples of the 14 patients with negative PB culture and no laboratory evidence of *Candida* spp. colonization. BDG was not detected in serum samples from this group (median 37.6 pg/mL, IQR 9.7 and 51.7), except for one very jaundiced serum sample, a condition known to cause false positive BDG results [7,44].

There was substantial concordance between the results of the qPCR assay and PB cultures (kappa value 0.76), while the concordance between the molecular assay and BDG test was only fair (kappa value 0.30).

## 4. Discussion

In this prospective study, a qPCR assay for the detection of the five major *Candida* species worldwide [5,45] was evaluated, with the aim of achieving a rapid and accurate diagnosis in critically ill patients suspected of *Candida* infection, compared to the gold standard method of blood cultures. We also proposed the use of SYBR™ Green reagent as a suitable option for resource-limited laboratories in developing countries [46].

Our qPCR assay succeeded in detecting *Candida* DNA in all 20 cases of positive PB cultures, indicating high accuracy in identifying true candidemia episodes. While direct comparisons with other published PCR techniques should be approached with caution due to potential differences in study designs, our assay outperformed previous in-house methods that showed low sensitivity values (ranging from 40% to 69%) [18,19,47,48]. Furthermore, our assay exhibited a similar sensitivity when compared with a preliminary study evaluating a multiplex qPCR for *Candida* DNA detection with beacon probes, which reported a positivity rate of 96% in patients with confirmed IC [49]. However, this promising result was not replicated in a recent prospective multicenter investigation that used the same multiplex PCR in serum and abdominal fluid samples from patients suspected of candidemia, as the multiplex PCR was positive in only 21.4% and 31.3% of respective samples from patients with positive blood cultures [50].

A number of commercially available assays for *Candida* DNA detection have been evaluated in recent studies, but they have shown variable positivity rates with samples from proven candidemia cases, ranging from 33% to 89% [26,29,35,38]. These results did not provide conclusive evidence for the superiority of commercial tests over in-house methods, as further evaluation and validation in clinical trials are necessary for the latter, as well as most commercial tests [24,26,27]. 

The ability of our molecular assay to identify four episodes of dual *Candida* infections that were missed by blood cultures is significant, particularly in critically ill patients. This is especially important when the missed species are potentially resistant to commonly used antifungal drugs, as observed in our study with the qPCR identification of *C. glabrata* and *C. krusei* as the second species in two and one samples, respectively [5,20,47]. 

In our study, BDG positivity was observed in all but one of the serum samples from the proven candidemia group. The exception was a sample with candidemia caused by *C. parapsilosis*, which was consistent with previous reports that have shown low levels of BDG in patients infected with *C. parapsilosis* [32,36,51]. 

The molecular assay also detected *Candida* species in six PB samples obtained from patients with CB-positive cultures. In the absence of positive PB cultures, however, we cannot rule out that the detection of *Candida* DNA in PB samples represents a transient candidemia [20,52]. However, we favor the likelihood of a diagnosis of true candidemia in these samples based on their high serum BDG levels, similar to those observed in the proven candidemia patients (medians of 457.0 and 478.1 pg/mL, respectively). In fact, intravascular catheter colonization by *Candida* species has most often been related to true candidemia episodes [52,53]. In contrast, the median BDG values in the 26 samples with CB-positive cultures but negative qPCR results was 131.2 pg/mL, which together may suggest either very low fungal burdens below the qPCR limit detection, or colonization of the catheters without bloodstream invasion [54].

Lastly, samples from patients who were neither infected nor colonized consistently yielded negative qPCR results. This was further supported by the concomitant negative results of the BDG test. These data strongly suggest that our qPCR assay was specific and could effectively rule out *Candida* infection. Previous reports have shown high predictive negative values of PCR assays in various nosocomial settings and in patients with diverse clinical conditions [11,27,55]. Nevertheless, the results of the molecular assay should be interpreted in the context of the clinical setting, to guide the discontinuation of empirical antifungal treatment [56,57].

This study has limitations. Our qPCR assay was designed to detect only five *Candida* species—although, they are major invasive species worldwide—which is also a limitation found in some commercial assays [23,24,26]. New species or less frequently isolated species may emerge in the nosocomial scenario, which would require customized assays [24,58]. In addition, the use of SYBR™ Green is not compatible with multiplex assays, whereas this would be possible by employing oligonucleotide probes. However, the lower cost of SYBR™ Green compared to species-specific probes makes our qPCR assay less expensive, even though it requires an individual reaction for each *Candida* species. On the contrary, the amplifications for detecting the five most important *Candida* species in the medical context can be performed simultaneously, as they use the same number of total cycles and the same cycling times and temperatures. 

Another limitation is that all patients included in this study underwent antifungal treatment after sample collection, regardless of the qPCR assay results. Thus, it was not possible to analyze any potential impact of these results on the patients’ outcomes.

In conclusion, our qPCR assay proved to be at least as sensitive as blood cultures, but with a shorter turnaround time. Additionally, it was able to detect a second *Candida* species in some samples, which was missed by the blood cultures. It also detected *Candida* DNA in some negative PB culture samples collected from patients at higher risk, as indicated by positive cultures for *Candida* species in CB samples. Importantly, our qPCR species identification was concordant with both PB and CB cultures, suggesting that it can be useful when used in parallel with blood cultures to facilitate earlier introduction of guided treatment instead of empirical treatment. Finally, our data suggest that a negative qPCR assay, together with a negative BDG result, provides evidence for the absence of hematogenous infection caused by the five major *Candida* species.

## Figures and Tables

**Table 1:** Demographic and clinical data of the 78 patients included in the study.

Variables	Patients
Age (years), median and range	60 (20–85)
Gender M/F	40 (51.6%)/38 (48.4%)
Underlying diseases	
Cancer	52 (66.6%)
Chronic/acute renal failure	8 (10.2%)
Others	18 (23.2%)
Candidemia risk factors	
Central venous catheter	78 (100.0%)
Previous *Candida* colonization	57 (73.1%)
Mechanical ventilation	35 (44.9%)
Dialysis	22 (28.2%)
Parenteral nutrition	25 (32.0%)
Previous antibiotic therapy (>96 h)	78 (100.0%)
Previous corticoid therapy	32 (41.0%)
SAPS3, median and range	64 (31–111)
ICU staying (days) prior to candidemia suspicion, median and range	12.5 (1–120)
30-day crude mortality	38 (48.7%)

SAPS3 = simplified acute physiology score; ICU = intensive care unit.

**Table 2:** Data of the samples with positive qPCR assay.

Patients’ Samples.	Blood Culture Isolation	qPCR Identification	Cq Values	BDG Values (pg/mL)
Samples with positive peripheral blood cultures				
S1	*Candida albicans*	*Candida albicans*	35.6	≥523
S2	*Candida glabrata*	*Candida glabrata*	20.1	≥523
S3	*Candida albicans*	*Candida albicans*	18.8	369.7
S4	*Candida albicans*	*Candida albicans/C. glabrata*	24.2/31.0	126.1
S5	*Candida glabrata*	*Candida glabrata*	20.8	433.2
S6	*Candida orthopsilosis*	*Candida parapsilosis* *	29.2	≥523
S7	*Candida tropicalis*	*Candida tropicalis*	35.4	≥523
S8	*Candida parapsilosis*	*Candida parapsilosis*	35.8	121.5
S9	*Candida parapsilosis*	*Candida parapsilosis*	20.7	193.3
S10	*Candida parapsilosis*	*Candida parapsilosis*	18.1	361.3
S11	*Candida tropicalis*	*Candida tropicalis*/*C. glabrata*	21.2/30.8	≥523
S12	*Candida tropicalis*	*Candida tropicalis*	22.1	≥523
S13	*Candida tropicalis*	*Candida tropicalis*	20.5	422.0
S14	*Candida albicans*	*Candida albicans*	21.1	≥523
S15	*Candida parapsilosis*	*Candida parapsilosis*	28.6	7.8
S16	*Candida glabrata*	*Candida glabrata*	19.6	≥523
S17	*Candida parapsilosis*	*Candida parapsilosis*	30.6	391.1
S18	*Candida albicans*	*Candida albicans*/*C. krusei*	23.0/27.1	≥523
S19	*Candida albicans*	*Candida albicans*	22.2	≥523
S20	*Candida albicans*	*Candida albicans*/*C. parapsilosis*	22.6/35.0	100.5
Samples with positive blood catheter cultures only				
S21	*Candida albicans*	*Candida albicans*	29.7	≥523
S22	*Candida albicans*	*Candida albicans*	28.9	424.4
S23	*Candida parapsilosis*	*Candida krusei*	23.0	157.0
S24	*Candida parapsilosis*	*Candida parapsilosis*	17.3	7.8
S25	*Candida glabrata*	*Candida glabrata*	17.8	457.0
S26	*Candida tropicalis*	*Candida tropicalis*	33.7	≥523

* qPCR assay identified *C. parapsilosis* complex; S4, S11, S18 and S20 = samples with. dual candidemia detected by qPCR assay.

## Data Availability

Data available on request from the corresponding author, due to ethical restrictions regarding patients’ data.

## Supplementary Materials

The following supporting information can be downloaded at: https://www.mdpi.com/article/10.3390/jof9060635/s1, Table S1: Primers employed in the qPCR assay for the identification and quantification of *Candida* species in blood samples.

## References

[1] Zhang S.X., Babady N.E., Hanson K.E., Harrington A.T., Larkin P.M., Leal S.M., Luethy P.M., Martin I.W., Pancholi P., Procop G.W. (2021). Recognition of Diagnostic Gaps for Laboratory Diagnosis of Fungal Diseases: Expert Opinion from the Fungal Diagnostics Laboratories Consortium (FDLC). J. Clin. Microbiol..

[2] Colombo A.L., Guimarães T., Sukienik T., Pasqualotto A.C., Andreotti R., Queiroz-Telles F., Nouér S.A., Nucci M. (2014). Prognostic factors and historical trends in the epidemiology of candidemia in critically ill patients: An analysis of five multicenter studies sequentially conducted over a 9-year period. Intensive Care Med..

[3] Doi A.M., Pignatari A.C., Edmond M.B., Marra A.R., Camargo LF A., Siqueira R.A., da Mota V.P., Colombo A.L. (2016). Epidemiology and Microbiologic Characterization of Nosocomial Candidemia from a Brazilian National Surveillance Program. PLoS ONE.

[4] Delaloye J., Calandra T. (2014). Invasive candidiasis as a cause of sepsis in the critically ill patient. Virulence.

[5] Pfaller M.A., Castanheira M. (2016). Nosocomial Candidiasis: Antifungal Stewardship and the Importance of Rapid Diagnosis. Med. Mycol..

[6] Kullberg B.J., Arendrup M.C. (2015). Invasive Candidiasis. N. Engl. J. Med..

[7] Pitarch A., Nombela C., Gil C. (2018). Diagnosis of Invasive Candidiasis: From Gold Standard Methods to Promising Leading-edge Technologies. Curr. Top. Med. Chem..

[8] Terrero-Salcedo D., Powers-Fletcher M.V. (2020). Updates in Laboratory Diagnostics for Invasive Fungal Infections. J. Clin. Microbiol..

[9] da Matta D.A., Souza A.C.R., Colombo A.L. (2017). Revisiting Species Distribution and Antifungal Susceptibility of *Candida* Bloodstream Isolates from Latin American Medical Centers. J. Fungi.

[10] Agnelli C., Valerio M., Bouza E., Guinea J., Sukiennik T., Guimarães T., Queiroz-Telles F., Muñoz P., Colombo A.L. (2022). Prognostic factors of *Candida* spp. bloodstream infection in adults: A nine-year retrospective cohort study across tertiary hospitals in Brazil and Spain. Lancet Reg. Health Am..

[11] Clancy C.J., Nguyen M.H. (2013). Finding the “missing 50%” of invasive candidiasis: How nonculture diagnostics will improve understanding of disease spectrum and transform patient care. Clin. Infect. Dis..

[12] Babb J., Clark A., Gaffney D., Abdelfattah K., Prokesch B.C. (2022). Little Utility of Fungal Blood Cultures in Surgical and Burn Intensive Care Units. Microbiol. Spectr..

[13] Mikulska M., Calandra T., Sanguinetti M., Poulain D., Viscoli C. (2010). The use of mannan antigen and anti-mannan antibodies in the diagnosis of invasive candidiasis: Recommendations from the Third European Conference on Infections in Leukemia. Crit. Care.

[14] Alves J., Alonso-Tarrés C., Rello J. (2022). How to Identify Invasive Candidemia in ICU-A Narrative Review. Antibiotics.

[15] Dupuis C., Le Bihan C., Maubon D., Calvet L., Ruckly S., Schwebel C., Bouadma L., Azoulay E., Cornet M., Timsit J.F. (2021). Performance of Repeated Measures of (1-3)-β-D-Glucan, Mannan Antigen, and Antimannan Antibodies for the Diagnosis of Invasive Candidiasis in ICU Patients: A Preplanned Ancillary Analysis of the EMPIRICUS Randomized Clinical Trial. Open. Forum Infect. Dis..

[16] Innings A., Ullberg M., Johansson A., Rubin C.J., Noreus N., Isaksson M., Herrmann B. (2007). Multiplex real-time PCR targeting the RNase P RNA gene for detection and identification of *Candida* species in blood. J. Clin. Microbiol..

[17] Lau A., Sorrell T.C., Chen S., Stanley K., Iredell J., Halliday C. (2008). Multiplex tandem PCR: A novel platform for rapid detection and identification of fungal pathogens from blood culture specimens. J. Clin. Microbiol..

[18] Avni T., Leibovici L., Paul M. (2011). PCR diagnosis of invasive candidiasis: Systematic review and meta-analysis. J. Clin. Microbiol..

[19] Schell W.A., Benton J.L., Smith P.B., Poore M., Rouse J.L., Boles D.J., Johnson M.D., Alexander B.D., Pamula V.K., Eckhardt A.E. (2012). Evaluation of a digital microfluidic real-time PCR platform to detect DNA of *Candida albicans* in blood. Eur. J. Clin. Microbiol. Infect. Dis..

[20] Taira C.L., Okay T.S., Delgado A.F., Ceccon M.E., de Almeida M.T., Del Negro G.M. (2014). A multiplex nested PCR for the detection and identification of *Candida* species in blood samples of critically ill paediatric patients. BMC Infect. Dis..

[21] Clancy C.J., Pappas P.G., Vazquez J., Judson M.A., Kontoyiannis D.P., Thompson G.R., Garey K.W., Reboli A., Greenberg R.N., Apewokin S. (2018). Detecting Infections Rapidly and Easily for Candidemia Trial, Part 2 (DIRECT2): A Prospective, Multicenter Study of the T2Candida Panel. Clin. Infect. Dis..

[22] Arvanitis M., Anagnostou T., Fuchs B.B., Caliendo A.M., Mylonakis E. (2014). Molecular and nonmolecular diagnostic methods for invasive fungal infections. Clin. Microbiol. Rev..

[23] Camp I., Spettel K., Willinger B. (2020). Molecular Methods for the Diagnosis of Invasive Candidiasis. J. Fungi.

[24] White P.L., Price J.S., Cordey A., Backx M. (2021). Molecular Diagnosis of Yeast Infections. Curr. Fungal Infect. Rep..

[25] Bassetti M., Azoulay E., Kullberg B.J., Ruhnke M., Shoham S., Vazquez J., Giacobbe D.R., Calandra T. (2021). EORTC/MSGERC Definitions of Invasive Fungal Diseases: Summary of Activities of the Intensive Care Unit Working Group. Clin. Infect. Dis..

[26] Clancy C.J., Nguyen M.H. (2019). Rapid diagnosis of invasive candidiasis: Ready for prime-time?. Curr. Opin. Infect. Dis..

[27] Kidd S.E., Chen S.C., Meyer W., Halliday C.L. (2019). A New Age in Molecular Diagnostics for Invasive Fungal Disease: Are We Ready?. Front. Microbiol..

[28] Mylonakis E., Clancy C.J., Ostrosky-Zeichner L., Garey K.W., Alangaden G.J., Vazquez J.A., Groeger J.S., Judson M.A., Vinagre Y.-M., Heard S.O. (2015). T2 magnetic resonance assay for the rapid diagnosis of candidemia in whole blood: A clinical trial. Clin. Infect. Dis..

[29] Arendrup M.C., Andersen J.S., Holten M.K., Krarup K.B., Reiter N., Schierbeck J., Helleberg M. (2019). Diagnostic Performance of T2Candida Among ICU Patients With Risk Factors for Invasive Candidiasis. Open. Forum Infect. Dis..

[30] White S.K., Schmidt R.L., Walker B.S., Hanson K.E. (2020). (1→3)-β-D-glucan testing for the detection of invasive fungal infections in immunocompromised or critically ill people. Cochrane Database Syst. Rev..

[31] Corcione S., Chasseur L., Lupia T., Shbaklo N., Scabini S., Filippini C., Mornese Pinna S., Morra di Celle S., Cavallo R., De Rosa F.G. (2022). The Diagnostic Relevance of β-D-Glucan for Candidemia within Internal Medicine Wards. Diagnostics.

[32] Mikulska M., Magnasco L., Signori A., Sepulcri C., Dettori S., Tutino S., Vena A., Miletich F., Ullah N., Morici P. (2022). Sensitivity of Serum Beta-D-Glucan in Candidemia According to *Candida* Species Epidemiology in Critically Ill Patients Admitted to the Intensive Care Unit. J. Fungi.

[33] Kritikos A., Poissy J., Croxatto A., Bochud P.Y., Pagani J.L., Lamoth F. (2020). Impact of the Beta-Glucan Test on Management of Intensive Care Unit Patients at Risk for Invasive Candidiasis. J. Clin. Microbiol..

[34] Kazancioglu S., Bastug A., Kayaaslan B., Mutlu N.M., Calci E., Turhan T., Mumcuoglu I., Akinci E., Bodur H. (2022). Diagnostic value of β-D-glucan alone or combined with Candida score, colonization index and C-reactive protein for candidemia. J. Infect. Dev. Ctries..

[35] Koc Ö., Kessler H.H., Hoenigl M., Wagener J., Suerbaum S., Schubert S., Dichtl K. (2022). Performance of Multiplex PCR and β-1,3-D-Glucan Testing for the Diagnosis of Candidemia. J. Fungi.

[36] Liu Y., Chen F., Zhu X., Shen L., Zhang S.X. (2015). Evaluation of a Novel Plasma (1,3)-β-d-Glucan Detection Assay for Diagnosis of Candidemia in Pediatric Patients. J. Clin. Microbiol..

[37] Gill C.M., Kenney R.M., Hencken L., Mlynarek M.E., Alangaden G.J., Samuel L.P., Davis S.L. (2019). T2 Candida versus beta-D-glucan to facilitate antifungal discontinuation in the intensive care unit. Diagn. Microbiol. Infect. Dis..

[38] McKeating C., White P.L., Posso R., Palmer M., Johnson E., McMullan R. (2018). Diagnostic accuracy of fungal PCR and β-d-glucan for detection of candidaemia: A preliminary evaluation. J. Clin. Pathol..

[39] Price J.S., Fallon M., Posso R., Backx M., White P.L. (2022). An Evaluation of the OLM *Cand*ID Real-Time PCR to Aid in the Diagnosis of Invasive Candidiasis When Testing Serum Samples. J. Fungi.

[40] de Almeida J.N., Sztajnbok J., da Silva A.R., Vieira V.A., Galastri A.L., Bissoli L., Litvinov N., Del Negro G.M.B., Motta A.L., Rossi F. (2016). Rapid identification of moulds and arthroconidial yeasts from positive blood cultures by MALDI-TOF mass spectrometry. Med. Mycol..

[41] Duffy T., Bisio M., Altcheh J., Burgos J.M., Diez M., Levin M.J., Favaloro R.R., Freilij H., Schijman A.G. (2009). Accurate real-time PCR strategy for monitoring bloodstream parasitic loads in chagas disease patients. PLoS Negl. Trop. Dis..

[42] Luciano P., Bertea C.M., Temporale G., Maffei M.E. (2007). DNA internal standard for the quantitative determination of hallucinogenic plants in plant mixtures. Forensic Sci. Int. Genet..

[43] Bustin S.A., Benes V., Garson J.A., Hellemans J., Huggett J., Kubista M., Mueller R., Nolan T., Pfaffl M.W., Shipley G.L. (2009). The MIQE guidelines: Minimum information for publication of quantitative real-time PCR experiments. Clin. Chem..

[44] Mennink-Kersten M.A., Ruegebrink D., Verweij P.E. (2008). *Pseudomonas aeruginosa* as a cause of 1,3-beta-D-glucan assay reactivity. Clin. Infect. Dis..

[45] Calandra T., Roberts J.A., Antonelli M., Bassetti M., Vincent J.L. (2016). Diagnosis and management of invasive candidiasis in the ICU: An updated approach to an old enemy. Crit. Care.

[46] Georgacopoulos O., Nunnally N.S., Le N., Lysen C., Welsh R.M., Kordalewska M., Perlin D.S., Berkow E.L., Sexton D.J. (2020). Performance Evaluation of Culture-Independent SYBR Green *Candida auris* Quantitative PCR Diagnostics on Anterior Nares Surveillance Swabs. J. Clin. Microbiol..

[47] Nguyen M.H., Wissel M.C., Shields R.K., Salomoni M.A., Hao B., Press E.G., Shields R.M., Cheng S., Mitsani D., Vadnerkar A. (2012). Performance of *Candida* real-time polymerase chain reaction, β-D-glucan assay, and blood cultures in the diagnosis of invasive candidiasis. Clin. Infect. Dis..

[48] Hasseine L., Cassaing S., Robert-Gangneux F., Fillaux J., Marty P., Gangneux J.P., Tattevin P. (2015). High negative predictive value diagnostic strategies for the reevaluation of early antifungal treatment: A multicenter prospective trial in patients at risk for invasive fungal infections. J. Infect..

[49] Fortún J., Meije Y., Buitrago M.J., Gago S., Bernal-Martinez L., Pemán J., Pérez M., Gómez-Gª Pedrosa E., Madrid N., Pintado V. (2014). Clinical validation of a multiplex real-time PCR assay for detection of invasive candidiasis in intensive care unit patients. J. Antimicrob. Chemother..

[50] Nieto M., Robles J.C., Causse M., Gutiérrez L., Cruz Perez M., Ferrer R., Xercavins M., Herrero E., Sirvent E., Fernández C. (2019). Polymerase Chain Reaction Versus Blood Culture to Detect *Candida* Species in High-Risk Patients with Suspected Invasive Candidiasis: The MICAFEM Study. Infect. Dis. Ther..

[51] Mikulska M., Giacobbe D.R., Furfaro E., Mesini A., Marchese A., Del Bono V., Viscoli C. (2016). Lower sensitivity of serum (1,3)-β-d-glucan for the diagnosis of candidaemia due to *Candida parapsilosis*. Clin. Microbiol. Infect..

[52] López-Medrano F., Fernández-Ruiz M., Origüen J., Belarte-Tornero L.C., Carazo-Medina R., Panizo-Mota F., Chaves F., Sanz-Sanz F., San Juan R., Aguado J.M. (2012). Clinical significance of *Candida* colonization of intravascular catheters in the absence of documented candidemia. Diagn. Microbiol. Infect. Dis..

[53] Fernández-Cruz A., Martín-Rabadán P., Suárez-Salas M., Rojas-Wettig L., Pérez M.J., Guinea J., Guembe M., Peláez T., Sánchez-Carrillo C., Bouza E. (2014). Is it feasible to diagnose catheter-related candidemia without catheter withdrawal?. Med. Mycol..

[54] Giacobbe D.R., Esteves P., Bruzzi P., Mikulska M., Furfaro E., Mesini A., Tatarelli P., Grignolo S., Viscoli C., Colombo A.L. (2015). Initial serum (1,3)-β-D-glucan as a predictor of mortality in proven candidaemia: Findings from a retrospective study in two teaching hospitals in Italy and Brazil. Clin. Microbiol. Infect..

[55] Arvanitis M., Ziakas P.D., Zacharioudakis I.M., Zervou F.N., Caliendo A.M., Mylonakis E. (2014). PCR in diagnosis of invasive aspergillosis: A meta-analysis of diagnostic performance. J. Clin. Microbiol..

[56] Kourkoumpetis T.K., Fuchs B.B., Coleman J.J., Desalermos A., Mylonakis E. (2012). Polymerase chain reaction-based assays for the diagnosis of invasive fungal infections. Clin. Infect. Dis..

[57] Pappas P.G., Kauffman C.A., Andes D.R., Clancy C.J., Marr K.A., Ostrosky-Zeichner L., Reboli A.C., Schuster M.G., Vazquez J.A., Walsh T.J. (2016). Clinical Practice Guideline for the Management of Candidiasis: 2016 Update by the Infectious Diseases Society of America. Clin. Infect. Dis..

[58] Lamoth F., Clancy C.J., Tissot F., Squires K., Eggimann P., Flückiger U., Siegemund M., Orasch C., Zimmerli S., Calandra T. (2020). Performance of the T2Candida Panel for the Diagnosis of Intra-abdominal Candidiasis. Open. Forum Infect. Dis..

