# Supplementary material for: Performance of a Real-Time PCR Assay for the Detection of Five Candida Species in Blood Samples from ICU Patients at Risk of Candidemia
Source: J Fungi (Basel). 2023 May 31;9(6):635. doi: 10.3390/jof9060635 (PMC10299639; doi:10.3390/jof9060635)
Supplement: Supplementary file 1 [file jof-09-00635-s001.zip › jof-2321788-supplementary.pdf]

Table S1. Primers employed in the qPCR assay for the identification and quantification of *Candida* species in blood samples

Primers	**Sequence (5' → 3')**	**Amplicons**	**References**
**CALB1/2**	F- GGGTTTGCTTGAAAGACGGTA<br>R-TTGAAGATATACGTGGTGGACGTTA	108 bp	(Guiver et al. 2001)
**CGL1/2**	F- GCGCCCCTTGCCTCTC<br>R-CCCAGGGCTATAACACTCTACACC	124 bp	(Brinkman et al., 2003)
**CTR1/2**	F- CAATCCTACCGCCAGAGGTTAT<br>R- TGGCCACTAGCAAAATAAGCGT	357 bp	(Luo and Mitchell, 2002)
**CPAR1/2***	F- GCCAGAGATTAAACTCAACCAA<br>R- CCTATCCATTAGTTTATACTCCGC	297 bp	(Luo and Mitchell, 2002)
**CKR1/2**	F- CTCAGATTTGAAATCGTGCTTTG<br>R- GGGGCTCTCACCCTCCTG	113 bp	(Brinkman *et al.*, 2003)
**GA2OX2**	F-GCTCGGCGACGAATGATTAC<br>R-CGGTGCTGGTGGATAGTGAT	-----	(Luciano et al., 2007)

CALB = *C. albicans*; CGL = *C. glabrata*; CTR = *C. tropicalis*; CPAR = *C. parapsilosis* complex; CKR = *C. krusei* ; GA2OX2 = *A. thaliana* primers (sequence of gibberellin 2-beta-dioxygenase); F = forward ; R = reverse; bp = base pairs
